# A Novel Perspective on the Proactive and Reactive Controls of Executive Function in Chronic Stroke Patients

**DOI:** 10.3389/fneur.2022.766622

**Published:** 2022-02-28

**Authors:** Qiuhua Yu, Xiaomin Huang, Baofeng Zhang, Zhicheng Li, Tao Zhang, Ziwei Hu, Minghui Ding, Zhenwen Liang, Wai Leung Ambrose Lo

**Affiliations:** ^1^Department of Rehabilitation Medicine, The First Affiliated Hospital, Sun Yat-sen University, Guangzhou, China; ^2^Department of Rehabilitation Medicine, Zhujiang Hospital, Southern Medical University, Guangzhou, China; ^3^School of Rehabilitation Medicine, Gannan Medical University, Ganzhou, China; ^4^Department of Rehabilitation Medicine, The First Affiliated Hospital of Jinan University, Guangzhou, China; ^5^Guangdong Engineering and Technology Research Centre for Rehabilitation Medicine and Translation, The First Affiliated Hospital, Sun Yat-sen University, Guangzhou, China

**Keywords:** executive function (EF), proactive control, reactive control, stroke, working memory

## Abstract

**Objectives:**

To investigate the proactive and reactive control process when executing a complex task in patients with stroke. Proactive control is the preparatory process before the target stimulus, whereas reactive control is an imperative resolution of interference after the target stimulus.

**Methods:**

In total, 17 patients with chronic stroke and 17 healthy individuals were recruited. The proactive and reactive control of executive function was assessed by the task-switching paradigm and the AX version of the Continuous Performance Task (AX-CPT). The general executive function was assessed by Color Trial Test (CTT) and Stroop Test. The behavioral data of the task-switching paradigm were analyzed by a three-way repeated-measures ANOVA, and the AX-CPT data were analyzed by two-way repeated-measures ANOVA.

**Results:**

For efficiency scores in the task-switching paradigm, trial (repeat vs. switch) × group (stroke or control group) interaction effect was significant. *Post-hoc* analysis on trial × group effect showed a significant between-trial difference in accuracy rates in the repeat trial in the control group regardless of 100 or 50% validity. For the AX-CPT, the main effects of condition and group on response time were statistically significant. The interaction effect of condition (AY or BX) × group (stroke or control group) was also significant. *Post-hoc* analysis for condition × group indicated that the stroke group had a significantly longer response time in the BX condition than the control group and longer completion time in CTT2 and larger word interference for completion time in the Stroop test than the control cohort.

**Conclusions:**

Post-stroke survivors showed deficits in the performance of proactive control but not in the performance of reactive control. Deficits in proactive control may be related to the impairment of working memory. Interventions that focus on proactive control may result in improved clinical outcomes.

## Introduction

Approximately 50–75% of chronic post-stroke survivors have mild-to-moderate cognitive dysfunction (Desmond, Moroney) (1). Executive functions are higher order of cognitive functions that consist of set-shifting, initiation, monitoring one's behavior, and self-regulating functions (2). Basic cognitive functions could be modulated and organized by executive functions to achieve goal-oriented behaviors (3). The research conducted by Laakso et al. (2) showed that ischemic stroke patients aged 55–85 years, who participated in the Helsinki (Finland) Stroke Aging Memory Study (SAM), had worse performance in executive functions assessments than healthy individuals, including the measures of set-shifting, initiation, response inhibition, and strategy formation. Executive function was considered to be the main factor that influenced the clinical outcome and the overall functional status in patients with stroke after rehabilitation (4). This subsequently contributes to limitations in performing daily activities and participating in social activities (2, 5). Therefore, the precise evaluation of executive function capacity in patients with stroke is essential.

The Montreal Cognitive Assessment (MoCA) is commonly adopted as a brief screening test of general cognitive function. Three subtests of the MoCA, which are the Trail Making B task, a phonemic fluency task, and a two-item verbal abstraction task, could be applied to assess executive functions (6). Executive dysfunctions are indicated if the patient could not complete the subtests. There are others specifically designed assessments to evaluate executive functions such as the Stroop test or the Color Trail Test (CTT) (2). A previous study showed that a large sample of stroke or brain tumor patients with lesions in the frontal region performed worse in the Stroop test than healthy individuals. This suggested an inhibition impairment in stroke patients who had unilateral frontal cortex lesions (7). However, the MoCA, Stroop test, and CTT do not consider the patients' capacity in different executive control processes, e.g., after a cue or target stimulus. Braver (2012) proposed the theory of dual mechanisms of control (DMC), which comprised of proactive and reactive controls. Proactive control is an early selection of goal-relevant information that is processed in a sustained manner of the working memory before the occurrence of the cognitively demanding events (8). It plays an important role in orienting the behavior before the event occurs in our daily life. On the contrary, reactive control refers to the immediate resolution of the current conflict or interference (8), especially when the conflict is without any preparation. The DMC model has been employed to explore the performance of executive function in a different sample population. For instance, published literature reported that elderly people and children tended to employ reactive control, while young adults were more reliant on proactive control in the response-compatibility task (9) and in the AX version of Continuous Performance Task (AX-CPT)(10). Thus, the DMC theory provides a better way to understand the flexibility of behavior regulation in complex situations in our daily life (8). However, most of the studies published to date explored executive dysfunctions without investigating the proactive and reactive controls in patients with chronic stroke.

Proactive control impairment is presented as the inability to employ the information conveyed by the cue to prepare for a response to resolve an upcoming conflict, which required working memory and sustain attention (8, 11). Execution of a functional movement requires an extent of motor anticipation (12), which was comparable to that in the proactive control of the DMC model (13). The results of a study that utilized an electroencephalogram to assess motor anticipation in patients with stroke indicated that motor anticipation impairment and hand motor function were moderately associated with motor planning (14). In Delphine et al.'s study, eight stroke patients were required to grasp and hold a brisk loading under predictive and reactive conditions. The onset time of grip force after the impact was significantly later in the paretic hand of stroke patients than in controls during both predictive and reactive conditions (15). The findings of these previous studies suggested that stroke patients have deficit in proactive control in a simple motor reaction task. This deficit may be related to the prominent impairments of motor task initiation and generation with anticipation loss (16). It remains unclear in terms of the behavioral regulation that involves proactive and reactive controls in patients with chronic stroke when executing a complex task that demands executive function.

This cross-sectional study aimed to explore the performance of proactive and reactive controls of executive functions in post-stroke survivors by the task-switching paradigm and AX-CPT. As impairment of working memory, sustained attention, and motor anticipation was previously reported in stroke patients (16–18), the hypothesis of the present study was that proactive control was more affected than reactive control when performing a complex functional task in patients with stroke. The findings of the present study extend the knowledge pool on executive dysfunction in patients with stroke and would be of interest to a wide audience.

## Methods

### Participants

Participants were recruited from the inpatient unit of the Department of Rehabilitation Medicine, the First Affiliated Hospital of the Sun Yat-sen University. All of the participants were screened by a trained doctor according to the following inclusion and exclusion criteria. Inclusion criteria of the stroke cohort were as follows: (1) age between 40 and 65 years old; (2) a time of at 3 months after stroke occurrence; (3) self-reported or informant-reported memory or cognitive complaints; (4) being right-handed. The exclusion criteria of the stroke cohort were as follows: (1) a MoCA score of ≤ 14; (2) severe sensorimotor impairment; (3) aphasia identified by the Western Aphasia Battery with an aphasia quotient <93.8; (4) a history of neurological or psychiatrist conditions prior to stroke occurrence; (5) participating in another intervention trial. Healthy participants who had matched age and education level were recruited as the control cohort. Written informed consent was obtained from the participants prior to enrollment. Ethical approval for the present study was granted by the Institutional Ethical Committee for Clinical Research and Animal Trials of the First Affiliated Hospital of Sun Yat-sen University (Approval No. [2020]073).

### Instruments and Experiment Design

The task-switching paradigm and AX-CPT were employed to assess the performance of proactive and reactive controls of executive function. The design of the task-switching paradigm and AX-CPT made reference to the published studies (13, 19). The general executive function was accessed by the CTT and the Stroop test. The task-switching paradigm and AX-CPT were conducted in the software E-prime 2.0 (Psychological Software Tools, Pittsburgh, PA, USA).

#### Task-Switching Paradigm

**Figure 1:**
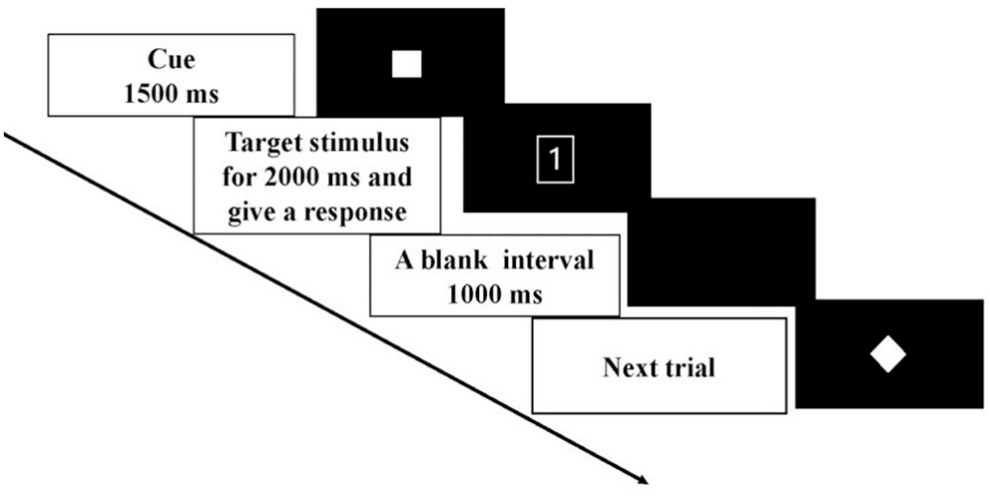
Schematic illustration of the time course of one trial in a task-switching paradigm.

In the task-switching paradigm, each trial began with a task cue (4 cm × 4 cm) at the center of the screen for 1,500 ms (Figure 1). The cue was followed by a target stimulus (4 cm × 4 cm) that lasted for 2,000ms. Participants were instructed to give a response to the target stimulus as soon as possible and within the 2,000 ms after the onset of the target stimulus. A blank interval of 1,000 ms then appeared, and the next trial began. There were two cue validities: 100 and 50%. The 100% valid cues were shown as a solid square (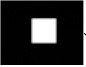) or a diamond (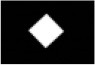). The 50% valid cue was shown as a solid star (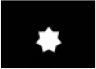). The cues with 100% validity asked the participants to prepare for the response selection rules based on the cue, whereas the cues with 50% validity did not convey any information about the following target stimulus. Thus, proactive and reactive controls were manipulated by the 100 and 50% validities cues, respectively. The ratio of the two types of cue validities was 1:1. The target stimulus formed by a digit (1 or 2) appeared inside the shape (a square or diamond) denoted two sets of response selection rules in this paradigm. One response selection rule was that a square with the digit “1” meant for the participant to press the button v on the computer keyboard, and a square with the digit “2” meant for the participant to press the button b. The other response selection rule was that the diamond with the digit “1” corresponded to the button b, and the diamond with the digit “2” corresponded to button v. In each trial, participants were required to make a response by following a rule that was different from (switch trial) or the same as (repeat trial) the previous trial. In the repeat trial, the response rule was the same as the previous trial, whereas in the switch trial the response rule was different from the previous one. The response time and accuracy rates were recorded during the experiment. There were two blocks and 61 trials for each block, in which the first trial was excluded for data analysis. It took around 6 min to complete one block followed by a 2–3 min break. The mixed block included the same ratio of both repeat (non-switch) trials and switch trials.

#### AX-CPT

**Figure 2:**
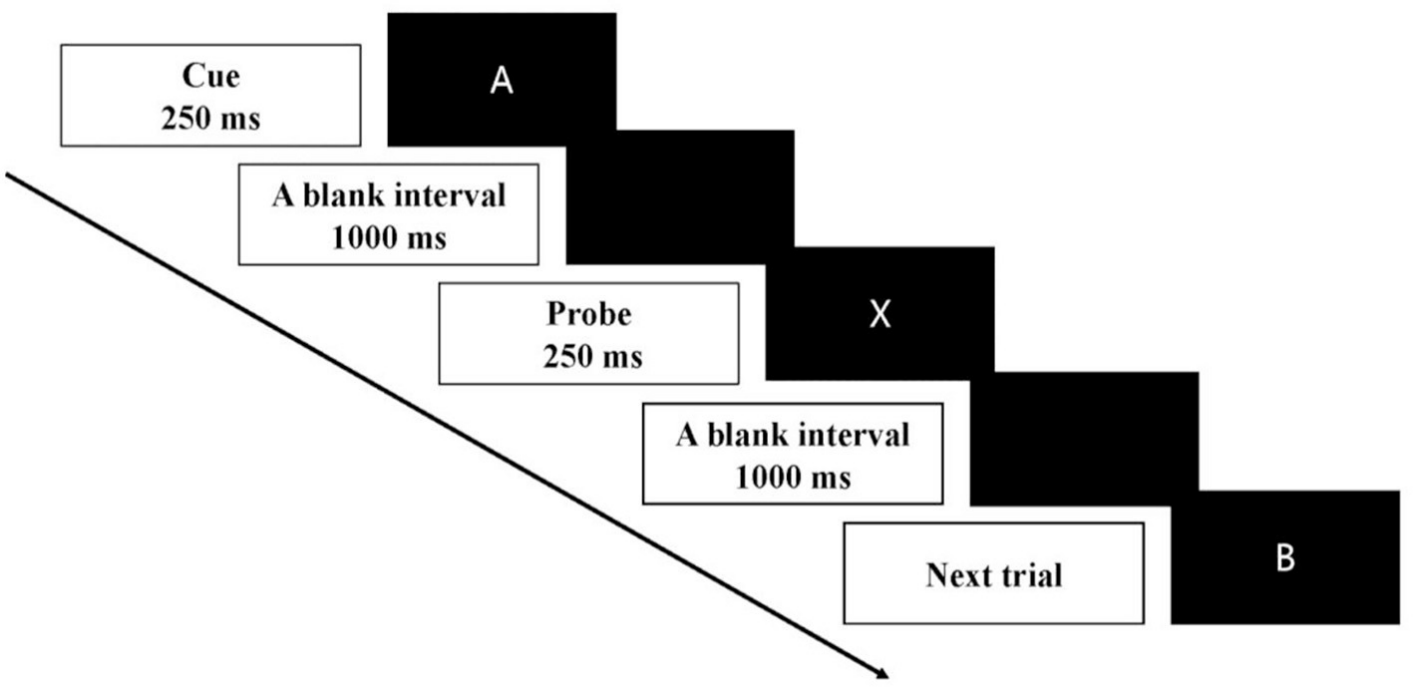
Schematic illustration of the time course of one trial in AX-CPT. Each trial of the AX-CPT began with a cue stimulus of 250 ms followed by a blank interval of 1,000 ms. The probe stimulus then appeared for 250 ms. After the onset of the probe stimulus, the participant was asked to give a response to the probe as soon as possible within 1,000 ms.

AX-CPT included four cue-probe pairs, which were A-X, A-Y, B-X, and B-Y. A or B served as a cue, and X or Y served as a probe (Figure 2). The cues (4 cm × 4 cm) and probe stimulus were presented at the center of the screen for 250 ms. A blank screen appeared in an inter-stimulus interval of 1,000 ms. After the onset of the probe stimulus, the participant was asked to give a response to the probe as soon as possible within the 1,000 ms. Participants were informed to press the button “v” on the keyboard as a target response for the probe “X” following the cue “A” and to press the button “b” as a non-target response for the other three cue-probe pairs. 70% of the trials were for AX condition, while 10% of the trials were for each of the other three conditions. The order of these four conditions was randomized. In total, there were two blocks, and each block was composed of 30 trials. It took approximately 2 min to complete one block with a short break between two blocks.

The AX-CPT was designed to assess the proactive control to maintain the cue in the working memory and reactive control to inhibit a prepotent response (20). Proactive control was primarily measured by the BX condition, in which adequate preparation elicited by the cue could enhance the reaction time for the probe. In contrast, reactive control was primarily measured by the AY condition, which required the greatest amount of reactive control to inhibit the prepotent motor response predicted by the cue of “A”.

#### Color Trail Test

CTT consisted of two parts (CTT1 and CTT2). For CTT1, numbered circles from 1 to 25 were randomly printed on a sheet of paper. Participants were asked to draw a line to join up all the circles in numerical order. CTT2 involved two circles for each number. One circle was in yellow and the other one was in red. Participants were required to draw a line to join up all the numbers from 1 to 25, alternating between the two colors (21). The time it took for completion, the number sequence, and color sequence errors were recorded by the examiner. An interference effect reflecting the shifting function was calculated by the completion time of CTT2 minus the completion time of CTT1 (Interference effect = CTT2 – CTT1).

#### Stroop Test

A Stroop test was performed to assess the function of conflict inhibition. The Stroop test consisted of two sub-tasks: naming color and naming the color in the printed words. The naming color task required participants to name the color patches printed in red, yellow, green, and blue colors. The task of naming color in printed word required participants to name the ink color of a set word, ignoring the words which were themselves the names of other colors. The 100 words or color patches of each sub-task were arranged in a 10 × 10 matrix. Participants were required to complete the subtasks correctly as quickly as they could. The response times it took for completion and the number of errors during each sub-task were recorded. The word interference of completion time, which is the completion time of naming colors minus the completion time of naming colors in printed words, was also included in the data analysis.

### Procedure

Background information (age, sex, height, weight, education experience, months after stroke, type of stroke, and lesion side and location) was recorded for all participants. In the task-switching paradigm and AX-CPT, the participant was seated in a quiet room in a relaxed position. A laptop computer for displaying the visual stimulus in the task-switching paradigm and AX-CPT was placed at a distance of 65–75cm in front of the participant. The task-switching paradigm and AX-CPT were randomly assigned to the participants. Before testing, the participant had the practice session to be familiar with each task. After finishing the task-switching paradigm and AX-CPT, the participant completed the MoCA, CTT, and Stroop test in a random order. Each participant completed all the tasks in two sessions (morning or afternoon) within 2 days. The task-switching paradigm and AX-CPT were conducted in one session which lasted for one hour. The MoCA, CTT, and Stroop test were conducted in the other session that lasted for around 1 h. Each participant was given sufficient time to rest between any two tasks.

### Data Analysis

Descriptive analysis was conducted to describe the characteristics of the sample populations. An independent *t*-test was applied to analyze the descriptive characteristics, the behavior data of CTT, Stroop test of the two groups of participants. A Chi-square test was conducted to analyze the between-group difference in sex. The Kolmogrov-Smirnov Test was conducted on the 24 variables of behavioral data to assess the normality of the data set. The result of the Kolmogrov-Smirnov Test showed that seven variables were significant (*p* < 0.050). The 17 variables of the data set were normally distributed. The behavioral data of the task-switching paradigm were analyzed by a three-way repeated-measures ANOVA: the within-subject factor was validity (100 or 50%) and trial (repeat or switch), and the between-subject factor was group (stroke or control group). The data of task switching paradigm showed significant correlations between participants' accuracy rates and reaction times in the control group (repeat trials with 100% cue validity: *r* = −0.693, *p* = 0.002; switch trials with 100% cue validity: *r* = −0.537, *p* = 0.026; repeat trials with 50% cue validity: *r* = −0.729, *p* = 0.001; switch trials with 50% cue validity: *r* = −0.517, *p* = 0.033). These significant correlations suggested a potential accuracy-speed trade-off, which would confound the results. To tackle the problem of accuracy-speed trade-off, the efficiency score (accuracy rate divided by reaction time) was used to analyze the behavioral data in each condition of the task-switching paradigm (22, 23). The behavior data of the AX-CPT were analyzed using a two-way repeated-measures ANOVA: the within-subject factor was condition (AY or BX), and the between-subject factor was group (stroke or control group). The dependent variables were the response time and accuracy rates in the AX-CPT and the response times, accuracy rates, and efficiency scores in repeat and switch trials in the task-switching paradigm. When significant main or interaction effects were observed in the two-way/three-way repeated-measures ANOVA, posthoc pairwise comparisons with the Bonferroni adjustment were applied. Pearson's correlation analysis was conducted to test the relationship between MOCA scores, general executive functions, and the performance in the task-switching paradigm and AX-CPT of the two cohorts. If multiple variables in the task-switching paradigm or AX-CPT were significantly related to general executive functions, a multiple regression model with a stepwise method was then conducted to verify the contributions of each variable to the capacity of general executive functions. All analyses were performed using the SPSS 20.0 (IBM SPSS Inc. Chicago, IL, USA) Windows software. The level of significance in the multivariate analyses was set at *p* < 0.05.

## Results

### Participants

In total, 17 participants with stroke (12 males and 5 females) and 17 control participants (8 males and 9 females) were recruited in this study. The sample characteristics of the two cohorts are presented in Table 1. The between-group difference in sex was not significant (*p* = 0.163). No significant between-group difference was shown in terms of age, weight, height, BMI, and education experience (see Table 1). The MoCA score in the stroke group was significantly lower than the control group (*p* < 0.001). Table 2 presents the clinical characteristics of each participant in the stroke cohort.

**Table 1:** Descriptive characteristics of the two groups of participants.

	**Stroke group**	**Control group**	***t***	***p***
	**Mean (SD)**	**Mean (SD)**		
Age (years)	53.82 (7.74)	52.18 (7.09)	0.647	0.522
Sex(male/female)^a^	12/5	8/9	1.943^b^	0.163
Weight (kg)	63.41 (9.03)	59.65 (10.93)	1.093	0.283
Height (m)	1.67 (0.05)	1.63 (0.08)	1.443	0.159
BMI	20.82 (3.16)	22.22 (3.21)	−1.280	0.210
Education experience (years)	11.53 (2.74)	11.65 (3.10)	−0.117	0.907
MoCA	22.12 (2.78)	26.82 (1.55)	−6.093	<0.001

a *Denotes chi square test for sex*,

b *Denotes Pearson chi-square value*.

**Table 2:** The clinical characteristics of the 17 chronic post-stroke participants.

**Participants**	**Sex**	**Age**	**Education level**	**Weight (kg)**	**Height (m)**	**BMI**	**Lesion side**	**Months after stroke**	**Type of stroke**	**Scores of MoCA**	**Lesion location**
1	Male	50	9	58	1.67	17.37	left	13	infarct	21	Infarction in right basal ganglia, frontal, temporal and insular lobes
2	Female	45	9	44	1.55	14.19	left	12	infarct	19	Right basal ganglia and radiata infarction
3	Male	65	9	70	1.68	20.83	left	25	infarct	21	Right basal ganglia and radiata infarction
4	Male	58	9	70	1.72	20.35	right	7	infarct	18	Right frontal lobe and pons infarction
5	Male	60	15	68	1.72	19.77	right	7	infarct	25	Left basal ganglia and radiata infarction
6	Male	48	15	70	1.7	20.59	left	12	hemorrhage	27	Hemorrhage in right basal ganglia
7	Male	41	9	78	1.72	22.67	left	5	infarct	22	Right basal ganglia and radiata infarction
8	Male	61	12	56	1.65	16.97	right	13	hemorrhage	22	Hemorrhage in left thalamus and left radiata infarction
9	Male	52	16	65	1.68	19.35	left	6	hemorrhage	25	Hemorrhage in right basal ganglia and radiata
10	Male	41	12	61	1.64	22.68	left	4	hemorrhage	26	Hemorrhage in left thalamus
11	Female	65	9	64	1.57	25.96	right	36	infarct	20	Right and left frontal lobe infarction
12	Male	52	12	60	1.68	21.26	left	25	hemorrhage	25	Hemorrhage in right putamen
13	Male	58	15	77.9	1.68	27.60	right	5	infarct	24	Left basal ganglia and radiata infarction
14	Male	62	9	70	1.76	22.60	left	4	infarct	20	Right basal ganglia infarction
15	Female	58	9	58	1.63	21.83	right	26	hemorrhage	21	Infarction in left frontal, parietal and insular lobes, and basal ganglia
16	Female	50	12	54	1.63	20.32	left	26	infarct	22	Right pons infarction
17	Female	49	15	54	1.66	19.60	left	7	hemorrhage	18	Infarction in left frontal, parietal and insular lobes, and basal ganglia

### Task-Switching Paradigm

Participants' accuracy rates of the repeat and switch trials in the task-switching paradigm are presented in Table 3. The proactive and reactive controls in the task-switching paradigm were manipulated by the 100 and 50% validities cues, respectively. The three-way repeated-measures ANOVA showed that the main effects of validity [*F*_(1, 32)_ = 1.319, *p* = 0.259, ηp^2^ = 0.040] and trial [*F*_(1, 32)_ = 1.315, *p* = 0.260, ηp^2^ = 0.039] were not statistically significant. However, the group main effect was statistically significant [*F*_(1, 32)_ = 12.174, *p* = 0.001, ηp^2^ = 0.276]. The interaction effect of validity × trial × group was not significant [*F*_(1, 32)_= 0.429, *p* = 0.517, ηp^2^ = 0.013]. Significant group main effect suggested that participants in the stroke group had lower accuracy rates than the controls regardless of validity and trial.

**Table 3:** Participants' accuracy rates (%) and response times (ms) of repeat and switch trials in task-switching paradigm stratified by group and validity.

**validity**	**Trial**	**Stroke group**		**Control group**	
		**Mean (SD)**		**Mean (SD)**	
		**ACC**	**RT**	**ACC**	**RT**
100%	Repeat	64.62 (21.74)	960.16 (160.42)	86.52 (14.37)	881.69 (139.55)
	Switch	58.79 (21.72)	1,084.89 (259.57)	75.40 (18.75)	1,103.36 (202.31)
50%	Repeat	59.34 (22.43)	1,003.37 (226.86)	83.87 (17.45)	981.95 (163.93)
	Switch	68.24 (17.41)	1,151.29 (244.53)	83.53 (14.55)	1,161.43 (153.97)

*100%, 100% valid cue; 50%, 50% valid cue; ACC, accuracy rate; RT, response time*.

Participants' response time of repeat and switch trials in task-switching paradigm are shown in Table 3. The main effects of validity [*F*_(1, 32)_ = 12.508, *p* = 0.001, ηp^2^ = 0.281] and trial [*F*_(1, 32)_ = 36.513, *p* < 0.001, ηp^2^ = 0.533] were significant. However, the group effect was not significant [*F*_(1, 32)_ = 0.097, *p* = 0.758, ηp^2^ = 0.003]. The interaction effect of validity × trial × group was not significant [*F*_(1, 32)_ = 0.1.186, *p* = 0.284, ηp^2^ = 0.036]. The interaction effect of trial × group was also not significant [*F*_(1, 32)_ = 1.328, *p* = 0.258, ηp^2^ = 0.040].

Participants' efficiency scores (accuracy rate divided by reaction time) of repeat and switch trials in the task-switching paradigm are shown in Table 4. The main effects of validity [*F*_(1, 32)_ = 1.444, *p* = 0.238, ηp^2^ = 0.043] were not statistically significant. However, the main effects of trial [*F*_(1, 32)_ = 23.352, *p* < 0.001, ηp^2^ = 0.422] and group were statistically significant [*F*_(1, 32)_ = 8.181, *p* = 0.007, ηp^2^ = 0.204]. The interaction effect of validity × trial × group was not significant [*F*_(1, 32)_= 0.014, *p* = 0.906, ηp^2^ < 0.001], whereas the trial × group interaction effect was significant [*F*_(1, 32)_ = 8.546, *p* = 0.006, ηp^2^ = 0.211]. A *post hoc* analysis of the trial × group effect on efficiency scores showed that no significant between-trial difference in efficiency scores in the stroke group (*p* = 0.187) in both 100 or 50% validity, whereas the efficiency scores in the repeat trial were significantly higher than the switch trial in the control group regardless of 100 or 50% validity (*p* < 0.001). Participants in the stroke group had lower efficiency scores than the controls in both repeat (*p* = 0.002) and switch trials (*p* = 0.068). These findings also suggested that the stroke group did not perform worse than the control group in the proactive and reactive controls of switching functions.

**Table 4:** Participants' efficiency scores (%/ms) of repeat and switch trials in task-switching paradigm stratified by group and validity.

**validity**	**Trial**	**Stroke group**	**Control group**
		**Mean (SD)**	**Mean (SD)**
100%	Repeat	0.069 (0.027)	0.103 (0.032)
	Switch	0.057 (0.022)	0.073 (0.030)
50%	Repeat	0.062 (0.027)	0.089 (0.025)
	Switch	0.063 (0.023)	0.074 (0.018)

*100%, 100% valid cue; 50%, 50% valid cue*.

### AX-CPT

The main effect of condition on accuracy rates was significant [*F*_(1, 32)_ = 8.196, *p* = 0.007, ηp^2^ = 0.204]. However, the group effect was not significant [*F*_(1, 32)_ = 3.344, *p* = 0.077, ηp^2^ = 0.095]. The interaction effect of condition × group was also not significant [*F*_(1, 32)_ = 0.719, *p* = 0.403, ηp^2^ = 0.022].

**Figure 3:**
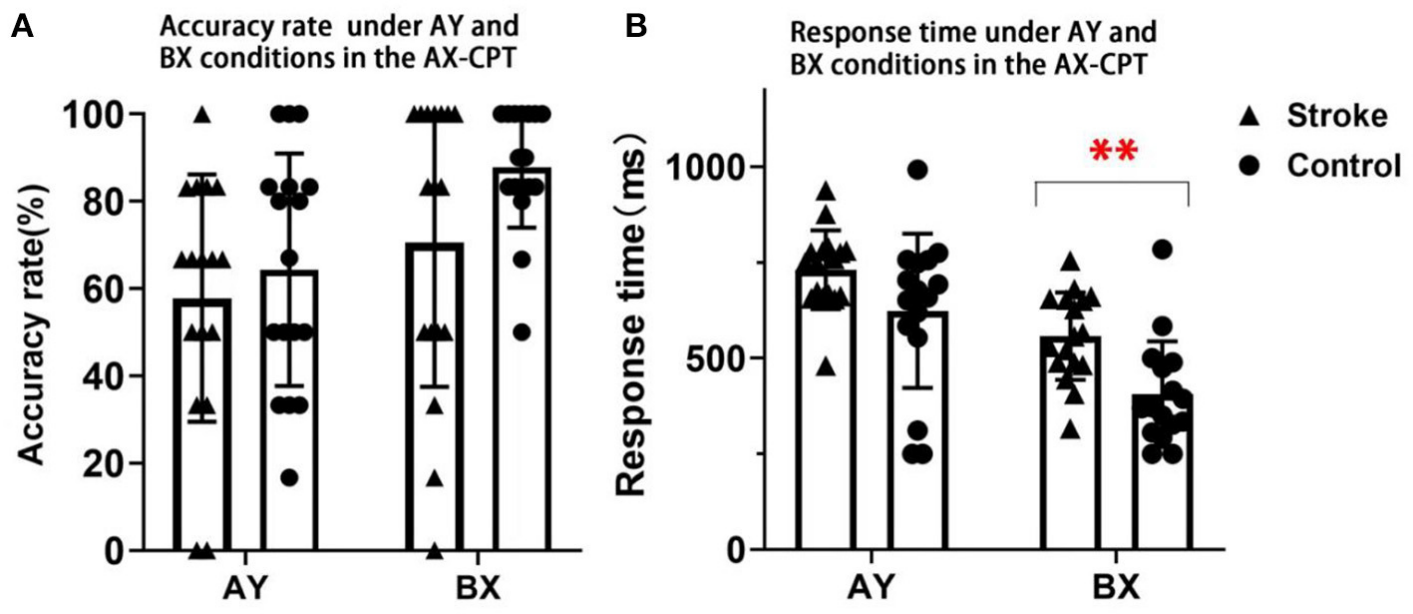
The accuracy rates and response time in AY and BX conditions of stroke and control participants in the AX-CPT. **(A)** Presents the results of accuracy rate. **(B)** Presents the results of response time. Triangles denote the single subject distribution of the stroke group. Dots denote the single subject distribution of the control group. Error bars denote +/– SD. ** Denotes *p* < 0.001.

The main effects of condition and group on response time were statistically significant, [*F*_(1, 32)_ = 61.151, *p* < 0.001, ηp^2^ = 0.656], [*F*_(1, 32)_ = 8.899, *p* = 0.005, ηp^2^ = 0.218]. The interaction effect of condition × group was also significant [*F*_(1, 32)_= 4.414, *p* = 0.044, ηp^2^ = 0.121] (Figure 3). *Post-hoc* analysis for condition × group indicated that the stroke participants had a significantly longer response time in the BX condition than the control group (*p* < 0.001) (Figure 3). No significant between-group difference in response time was found in the AY condition (*p* = 0.169). These findings suggested that the proactive control that was related to working memory was impaired in patients with stroke.

### Color Trail Test

No significant between-group difference was observed in the error rates of CTT1 and CTT2 (*p* > 0.050). However, participants in the stroke group showed significantly longer completion time in CTT1 (*p* = 0.002), CTT2 (*p* < 0.001) and a larger interference effect (*p* = 0.019) than the control group (Table 5).

**Table 5:** The error rates and response times in the color trail test.

	**Stroke group**	**Control group**	***t***	***p***
	**Mean (SD)**	**Mean (SD)**		
CTT1 NER(%)	1.65 (3.76)	1.18 (2.74)	0.417	0.679
CTT2 NER(%)	0.24 (0.97)	0.47 (1.94)	−0.447	0.658
CTT2 CER(%)	0.71 (2.11)	0.00 (0.00)	1.376	0.188
CTT1 CT(s)	127.71 (68.54)	65.24 (18.28)	3.631	0.002
CTT2 CT(s)	179.18 (65.33)	90.06 (24.17)	5.275	<0.001
Interference effect	51.47 (41.52)	24.82 (15.87)	2.472	0.019

*NER, number sequence error rates; CER, color sequence error rates; CT, completion time*.

### Stroop Test

The error rates and the self-corrected error rates showed no significant between-group difference in naming color and naming the color of the printed word of the Stroop test (*p* > 0.050). However, the stroke group showed a significantly longer completion time in naming the color (*p* = 0.002), naming the color of the printed word (*p* = 0.001), and the word interference of CT (*p* = 0.029) (Table 6).

**Table 6:** The error rates and response times in the stroop test.

	**Stroke group**	**Control group**	***t***	***p***
	**Mean (SD)**	**Mean (SD)**		
ER in naming colors (%)	1.35 (2.06)	0.41(0.94)	1.714	0.100
ER in naming colors in printed words (%)	3.82 (5.99)	1.24 (2.17)	1.675	0.104
CT in naming colors (%)	125.88 (49.81)	82.35 (18.09)	3.387	0.002
CT in naming colors in printed words (%)	217.06 (65.21)	133.06 (68.76)	3.655	0.001
Word interference of CT	91.18 (38.09)	50.71 (62.07)	2.291	0.029

*ER, error rates; SER, self-corrected error rates; CT, completion time*.

### The Relationship Between the MOCA Scores, General Executive Functions, and Proactive and Reactive Controls in the Two Cohorts

The relationship between general executive functions and proactive and reactive controls of the two groups are presented in Table 7. In the task-switching paradigm, the stroke group showed negative correlations between the accuracy rates of any trial type under 100 and 50% validities and the interference effect of CTT (*r* = −0.494 to −0.507, *p* < 0.050). Thus, the accuracy rates of repeat and switch trials under both 100 and 50% validities were put into the multiple regression model as the independent variables for the stroke group. The results of multiple regression analysis showed that only the accuracy rate of the switch trials under the 50% validity condition was a significant factor (*R*^2^ = 0.326, *B* = −0.571, *p* = 0.017). However, the accuracy rate of the switch trials under the 50% validity was only correlated with the interference effect in CTT in the control group (*r* = −0.614, *p* = 0.009). No significant association was found between the outcome variables in the task-switching paradigm and the word interference in the Stroop test (*p* > 0.050). The associations between MOCA scores and accuracy rates/response time of any trial type under 100 and 50% validities in two cohorts were also not significant (*p* > 0.050).

**Table 7:** The relationship between the general executive functions and the performance in the task-switching paradigm and AX-CPT of the two groups of participants.

	**Interference**		**Word interference of**	
	**effect of CTT**		**CT of stroop test**	
	**Stroke**	**Control**	**Stroke**	**Control**
100%_R_ACC	−0.494*	−0.213	−0.219	0.016
100%_S_ACC	−0.497*	0.005	−0.195	−0.172
50%_R_ACC	−0.507*	−0.191	−0.303	0.188
50%_S_ACC	−0.571*	−0.614**	−0.420	0.335
100%_R_RT	0.071	0.155	0.314	0.172
100%_S_RT	−0.132	0.267	−0.278	−0.206
50%_R_RT	−0.079	0.267	0.235	−0.117
50%_S_RT	0.130	0.045	−0.008	0.025
AY_ACC	0.028	0.098	0.279	−0.189
BX_ACC	−0.130	0.175	−0.430	−0.133
AY_RT	−0.035	−0.403	0.347	0.675**
BX_RT	0.206	−0.179	0.347	0.829**

The interference effect of CTT is calculated by the completion time of CTT2 minus the completion time of CTT1 (Interference effect = CTT2 – CTT1). The word interference of completion time is the completion time of naming colors minus the completion time of naming colors in printed words. Keys: 100%, 100% validity; 50%, 50% validity; R, repeat; S, switch; ACC, accuracy rates; RT, response time; CT, completion time; AY, AY condition; BX, BX condition. *p < 0.05; **p < 0.01.

In the AX-CPT, there was no significant correlation between the outcome variables in the AX-CPT, the interference effect in CTT, and MOCA scores in the two groups (*p* > 0.050). Significant correlations were found between the word interference of the Stroop test and the response time in AY (*r* = 0.675, *p* = 0.003) and BX (*r* = 0.829, *p* < 0.001) conditions in the control group but not in the stroke group. Thus, the response time of AY and BX conditions were put into the multiple regression model as the independent variables for the control group. The results of multiple regression analysis showed that only the response time in the BX condition was a significant factor (*R*^2^ = 0.688, *B* = 0.829, *p* < 0.001), whereas the response time in the AY condition was not significant (*p* = 0.433). These results further confirmed that stroke patients had impaired proactive control, which was related to conflict monitoring.

## Discussion

The present study investigated the performance of proactive and reactive controls of executive functions in post-stroke survivors by the dual cognitive control model. The main findings indicated that post-stroke participants require longer response times in the BX condition of the AX-CPT, but did not exhibit a reduction in the proactive and reactive control of switching compared with controls during the task-switching paradigm. These novel findings supported the hypothesis that proactive control was more affected than reactive control when performing a complex functional task.

In the BX condition, participants needed to maintain the “B” cue in the working memory, so that adequate preparation, which could enhance the reaction time for the probe (20). This study found that the post-stroke participants required a longer response time in the BX condition of the AX-CPT, suggesting that proactive control was impaired in post-stroke survivors. This finding was supported by two other previous studies that explored the proactive and reactive controls without involving executive functions in stroke patients (15, 24). One study showed impairment in the feed-forward adjustments of the multi-finger synergies prior to quick action in patients after a single cortical stroke with mild motor impairments (24). Dispa et al. found that the onset time of grip force after the impact was significantly later in the paretic hand of stroke patients than in controls in both predictive and reactive conditions (15). These findings suggested that stroke patients had deficits in proactive control in a motor reaction task. In the present study, the worse performance in proactive control of AX-CPT observed in the stroke group maybe resulted from the impairment of working memory, sustained attention, and anticipation loss. The execution of a complex task places a high demand on cognitive resources, including working memory, anticipation, and sustained attention during the proactive control period. The potential reasons for the worse performance in the proactive control in the stroke group are as follows. First, proactive control in AX-CPT was reported to be reliant upon the anticipation and prevention of interference before it occurred in the working memory (8, 20). Several studies have indicated working memory deficits in patients with stroke (18, 25). In the study by van Geldorp et al. (18), the participants were required to remember which cue was presented after a short temporal delay in a computerized delayed-match-to-sample task which included spatial, object, or binding (spatial + object) cues. Compared to healthy controls, the stroke participants showed working memory deficits in the conditions of spatial and object cues. Second, the impaired proactive control might be related to the anticipation loss in stroke patients. A study conducted by Roussel and Martinaud (16) indicated that in post-stroke participants, the dysexecutive profile was characterized by prominent impairments of motor initiation and generation with anticipation loss in the behavioral domain. In addition, several studies reported that proactive control is a sustained top-down attentional control (11, 13, 26). Manard et al. found that impaired proactive control of elderly people was related to a reduction in sustained neural activity at the frontal cortex (26). The sustained neural activity was related to sustained attention (11, 13). Patients with stroke were reported to have sustained attention deficits (17, 27), which could reduce the ability to expect and prepare for an upcoming stimulus (28). Therefore, deficits in sustained attention are likely to be a contributing factor to the impaired proactive control of stroke patients. However, previous studies indicated that motivation, fatigue, and mind wandering might have a negative effect on sustained attention for elderly people (29). Proactive control generally requires more effort, subsequently leads to more fatigue, which is most likely associated with the tendency to favor reactive over proactive control. Even though the participants in this study rested between any two tasks, it could not be ascertained that whether motivation, fatigue, and mind wondering might affect the behavior performance of proactive control. A future study that investigates the effect of these factors on the behavioral performance of proactive control would be beneficial to substantiate the findings of the present study.

According to the results of the task-switching paradigm, the stroke group had lower accuracy rates and efficiency scores in both repeat and switch trials than the control group. The two cohorts, however, did not show the between-trial difference in the 100 and 50% validities, which may suggest participants in the stroke group did not perform worse than healthy participants in the proactive and reactive controls of set-shifting function. These findings were consistent with the previous study, which demonstrated patients with stroke had different switch costs of accuracy rates compared with the control group in alternating switch tasks, but not in the cued task-switching (30). The findings in Pohl et al.'s study suggested that task switching in alternating switch tasks with a high demand of endogenous proactive control was impaired in adults after stroke, whereas task switching in the cued task-switching requiring exogenous proactive control was not impaired. The first reason was likely due to the higher difficulty level under endogenous proactive control than under exogenous proactive control. The second reason may be that the key neural structures that are responsible for the proactive and reactive control in task switching are different. Proactive control involves the frontal and/or parietal cortices (13, 20), whereas a wider network of brain regions are responsible for the reactive control in task switching, which involved the dorsolateral–ventrolateral plane of the prefrontal cortex and anterior cingulate cortex (ACC) (31, 32). However, both the present study and Pohl et al.'s study did not consider the different lesion locations between the two cohorts. This might potentially contribute to the lack of observed difference between the proactive and reactive controls of switching function in the stroke group.

The efficiency scores in the task-switching paradigm showed no significant between-trial difference in the stroke group, whereas the efficiency scores in the repeat trial of the control group were significantly higher than those in the switch trial regardless of 100 or 50% validity. These findings suggested that post-stroke survivors were less susceptible to proactive interference, which may be due to their lower working memory function. Allport and colleagues assumed that switch costs arose from proactive interference (33). Higher susceptibility of proactive interference in task switching was associated with higher working memory capacity (34, 35). Several studies had indicated working memory deficits in post-stroke survivors (18, 36), which was likely to be a factor that contributed to lower susceptibility to proactive interference in post-stroke survivors than healthy individuals. Impaired proactive control in the AX-CPT of stroke patients also supported deficits in the working memory since proactive control in the AX-CPT was related to working memory (20).

A larger interference effect in CTT and higher word interference of completion time in Stroop test were observed in stroke participants compared with healthy controls. The interference effect in CTT assessed the shifting function (24), while the word interference of completion time in the Stroop test taps on the conflict inhibition during the naming color task (37). The results of the CTT and Stroop test suggested that the general executive function was impaired in stroke participants. These findings were consistent with a previous study (2), which showed that stroke patients performed poorly in the Trail Making test and Stroop test. The results of the correlation and regression analysis between the Stroop test and the performance in BX condition of AX-CPT of the two groups further supported the notion that the proactive control for working memory function was impaired in post-stroke survivors. The process of interference control in the Stroop test included the top-down control process (38), which was also required during proactive control in AX-CPT (26). These two previous studies reported that the prefrontal cortex was the key node activated in the top-down control process in the Stroop test and proactive control. Thus, the response time in the BX condition related to proactive control was a significant factor related to the performance in the Stroop test.

## Limitations

There are several limitations in the present study that limit the interpretation of the data. First, the sample size was not power calculated and was likely to contain type II error. This might contribute to the lack of between-group differences in the task-switching paradigm. Second, the effects of lesion locations or lesion size on the proactive and reactive controls were not considered in the present study. Future study should consider recruiting sample group that has homogeneous lesion site and size. Third, the data related to the lesion such as side, temporal proximity, and the extent of the lesion were not taken into consideration in the present study. Future studies should consider the lesion data to further assess the impact on behavioral data. Forth, the sample population of the present study was heterogeneous which limits the interpretation of the findings. Fifth, two-way/three-way repeated-measures ANOVA applied to non-parametric data might lead to Type-I error. Sixth, the present study only employed a simple algorithm to explore the between-group difference in the speed-accuracy trade-off. Future studies should employ the diffusion drift models or other methodologies for in-depth exploring the between-group difference in the behavioral responses. In addition to behavioral outcomes, future research could adopt electrophysiology and brain-imaging techniques to investigate the underlying neural mechanisms (39). Last but not least, other research results showed a significant gender difference in proactive and reactive controls (40). Thus, there might be gender differences in proactive and reactive control performance of stroke patients. Further studies are required to clarify the gender effect on the performance of proactive and reactive controls in post-stroke survivors.

## Conclusion

Proactive control is the preparatory process before the target stimulus, whereas reactive control is an imperative process of interference resolution after the target stimulus. Post-stroke survivors showed a deficit in the performance of proactive control that is related to working memory. However, post-stroke patients seemed not to show a significant deficit in the reactive control of executive functions. Interventions that focus on proactive control may result in improved clinical outcomes.

## Data Availability Statement

The raw data supporting the conclusions of this article will be made available by the authors, without undue reservation.

## Ethics Statement

The studies involving human participants were reviewed and approved by the Institutional Ethical Committee for Clinical Research and Animal Trials of the First Affiliated Hospital of Sun Yat-sen University (Approval No. [2020]073). The patients/participants provided their written informed consent to participate in this study.

## Author Contributions

All authors meet the four primary ICMJE criteria for authorship. In addition, all authors have been actively involved in the study in different capacities: QY and WL: designed the study and conducted all stages of the study including data collection, analysis, interpretation, and drafting of the manuscript. XH, ZH, BZ, and ZLi: participated in the design of the research protocol, data analysis, and drafting of the manuscript. TZ, ZH, MD, and ZLia: participated in data collection, analysis, and interpretation of the data. WL and ZLia: revised the manuscript, interpreted the data, and managed the trial. All authors have read and approved the final manuscript.

## Funding

This research project was supported by the National Natural Science Foundation of China (Grant Numbers: 81971224 and 82002375); Science and Technology Program of Guangzhou (Grant Number: 201803010083).

## Conflict of Interest

The authors declare that the research was conducted in the absence of any commercial or financial relationships that could be construed as a potential conflict of interest.

## Publisher's Note

All claims expressed in this article are solely those of the authors and do not necessarily represent those of their affiliated organizations, or those of the publisher, the editors and the reviewers. Any product that may be evaluated in this article, or claim that may be made by its manufacturer, is not guaranteed or endorsed by the publisher.
